# Recurrent Pericarditis in Children and Adolescents

**DOI:** 10.3389/fped.2019.00419

**Published:** 2019-10-18

**Authors:** Enrico Tombetti, Teresa Giani, Antonio Brucato, Rolando Cimaz

**Affiliations:** ^1^Department of Medicine, Azienda Socio Sanitaria Territoriale (ASST) Fetebenefratelli-Sacco and Department of “Biomedical and Clinical Sciences Luigi Sacco”, Milan University, Milan, Italy; ^2^Rheumatology Unit, Department of Pediatrics, Anna Meyer Children's Hospital, University of Florence, Florence, Italy; ^3^Department of Medical Biotechnology, University of Siena, Siena, Italy; ^4^Department of Clinical Sciences and Community Health, University of Milan, Milan, Italy; ^5^Azienda Socio Sanitaria Territoriale (ASST) G.Pini, Milan, Italy

**Keywords:** pericarditis, myopericarditis, children, adolescents, pediatric, autoinflammatory diseases

## Abstract

Recurrent pericarditis (RP) is a clinical syndrome characterized by recurrent attacks of acute pericardial inflammation. Prognosis *quoad vitam* is good, although morbidity might be significant, especially in children and adolescents. Multiple potential etiologies result in RP, in the vast majority of cases through autoimmune or autoinflammatory mechanisms. Idiopathic RP is one of the most frequent diagnoses, that requires the exclusion of all known etiologies. Therapeutic advances in the last decade have been significant with the recognition of the effectiveness of anti IL1 therapy, but a correct diagnostic and therapeutic algorithm is of key importance. Unfortunately, most of evidence comes from studies in adult patients. Here we review the etiopathogenesis, diagnosis and management of RP in pediatric patients.

## Pericarditis: Definitions

Pericarditis is a clinical syndrome characterized by pericardial inflammation with or without concurrent pericardial effusion.

According to the European Society of Cardiology (ESC) guidelines (1), diagnosis of acute pericarditis requires at least two of the objective criteria listed in Table 1. The clinical course is distinguished into acute, incessant, recurrent and chronic pericarditis by temporal cut-offs defined by expert consensus (Table 1). Acute pericarditis accounts for 5% of the presentations to the emergency department for chest pain in pediatric patients (3). After the attack has subsided, acute pericarditis may recur leading to recurrent pericarditis (RP) in about 15–30% of adult patients (4, 5) and in 35% of pediatric patients (6). Recurrences are frequently less severe than the first attack.

**Table 1:** Definition and diagnostic criteria for pericarditis.

	**Definition and diagnostic criteria**
Acute Pericarditis	Acute (lasting <4–6 weeks) inflammatory pericardial syndrome to be diagnosed by ≥2 of the following: Pericardial chest pain^*^ (prevalence in pediatric cases 90–95%) Pericardial rubs (prevalence in pediatric cases 30%) New widespread ST-elevation or PR depression on ECG (prevalence in pediatric cases 40–50%) Pericardial effusion (new or worsening, prevalence in pediatric cases 70–80%) Additional supporting findings: - Elevated inflammatory markers (e.g., C-reactive protein, erythrocyte sedimentation rate, and white blood cell count) - Pericardial inflammation at imaging (CT, CMR)
Recurrent pericarditis	Recurrence of acute pericarditis after a documented first episode and a symptom-free interval of ≥4–6 weeks

*Adapted from Adler et al. (1) and Imazio et al. (2)*.

*CT, computed tomography; ECG, electrocardiogram; CMR, cardiac magnetic resonance*.

* *Pericardial chest pain: typically sharp and with pleuritic features; improved by sitting and leaning forward*.

Most of evidence about pericarditis comes from studies on adults. However, RP in children and adolescents is frequent, and has important specificities that will be review here.

## Etiology, Epidemiology, and Diagnosis

### Clinical and Etiological Classification

Acute pericarditis recognizes multiple etiologies, including infections, autoimmunity, autoinflammation, genetic abnormalities, drugs, cardiac injuries, and undetermined causes resulting in idiopathic pericarditis (Table 2). Most frequent etiologies show geographical variation and depend on the clinical course. Worldwide, tuberculosis is the most common cause of pericarditis. In developed countries, a viral infection is the most common cause of the first pericarditis attack, while idiopathic RP (IRP) accounts for about 80% of adults and 70% of children with RP.

**Table 2:** Most common etiologies of acute pericarditis in the pediatric age.

Infectious^#^	Purulent – pyogenic bacteria Tubercular Viral^*^ (adenovirus, enterovirus, parvovirus, influenza A, cytomegalovirus, Epstein-Barr Virus, human herpesvirus-6) Other: Lyme's disease, *Histoplasma capsulatum*, and opportunistic infections in immunocompromised patients
Autoimmune	Connective tissue diseases^*^ (systemic lupus erythematous [SLE], dermatomyositis) Arthritis (Systemic-onset juvenile idiopathic arthritis [so-JIA]^*^, rheumatic fever^*^) Vasculitis^*^ (Takayasu, Kawasaki, Behçet disease and ANCA-associated vasculitides) Sarcoidosis^*^ Inflammatory bowel diseases^*^
Autoinflammatory	Familiar Mediterranean Fever (FMF)^*^ TNF receptor-associated periodic syndrome (TRAPS)^*^
Other genetic conditions	Camptodactyly–arthropathy–coxa vara– pericarditis (CACP) syndrome
Iatrogenic	Chemotherapy- and radiotherapy- related Immune checkpoint-inhibitors (7) Drug-related SLE (8) Hypersensitivity (e.g., penicillins, mesalamine, sulfasalazine, infliximab)
Post-cardiac injury	Myocardial infarction, pericardiotomy^*^, pericardial bleeding^*^, percutaneous coronary intervention^*^, pacemaker lead insertion^*^, radiofrequency ablation^*^, chest trauma^*^
Miscellaneous	Malignancies Uremia Mixedema Anorexia nervosa
Idiopathic	^*^

* *Potential evolution to recurrent pericarditis*.

# *Potentially favored by inherited or acquired immune deficiencies*.

### Idiopathic Recurrent Pericarditis (IRP)

Limited epidemiologic data are available for pediatric IRP. Acute idiopathic pericarditis equally affects male and female children, while being more prevalent in male among adolescents (9). The reported incidence in the general population of acute pericarditis is 30–150/10^5^ per year (10, 11). Considering the probability of recurrence and of alternative etiologies, the incidence of IRP can be estimated at about 5–35/10^5^ per year. After the first recurrence, up to 50% of patients undergo further pericarditis attacks (12, 13).

IRP is a diagnosis of exclusion, that can be made only after an exhaustive screening. Invasive procedures (e.g., pericardiocentesis and pericardial biopsy) allow to increase the sensitivity for specific etiologies (14), but are rarely performed and there is no consensus about which tests should be performed before concluding for IRP.

Despite that the term “idiopathic” reflects our ignorance about the etiology of these conditions, recent advances have allowed an increased awareness about the autoimmune/autoinflammatory pathogenesis and about patients heterogeneity. In our experience, there are three extreme phenotypes within IRP:

- Recurrent attacks of pericarditis followed by complete resolution with highly symptomatic serositis, high fever and strikingly elevated acute-phase reactants. This phenotype is particularly frequent in pediatric cases (2) and typically shows a spectacular response to anti-interleukin-1 (IL1) therapies such as anakinra (15). Important similarities with autoinflammatory conditions (see below), suggests a similar pathogenesis.
- Recurrent attacks with a subacute course, moderate to high elevation of acute-phase reactants, frequent autoantibody positivity (anti-nuclear antibodies, ANA, anti-heart antibodies, AHA, and anti-intercalated disk autoantibodies) and presence of other features occurring in systemic autoimmune diseases (e.g., arthralgias, xeroftalmia, Raynaud's phenomenon, discoid lupus, uveitis). Autoimmune mechanisms are believed to play an important role in these patients. However, we highlight that autoantibodies are not a specific markers of an autoimmune pathogenesis, as they may be an epiphenomenon of pericardial inflammation.
- Patients with mild attacks with a subacute or grumbling course, smoldering elevation of inflammatory markers, and no evidence of autoimmunity.

### Post-cardiac Injury Recurrent Pericarditis

Post-cardiac injury syndromes are characterized by pleuro-pericarditis occurring after myocardial infarction, chest trauma, cardiac surgery, and percutaneous procedures including angioplasty/coronary stenting, cardiovascular implantable electronic device lead insertion and radiofrequency ablation (16–18). Among these, post-pericardiotomy (PP) pericarditis is the best-characterized condition. PP pericarditis occurs after heart surgery in about 15–30% of subjects (19, 20), typically within 3 months. The observed risk to develop RP after the first attack ranges between 1 and 2% in the studies performed in the last decade (19, 20) and 50% in those performed in the early 90s' (50%) (21).

It is debated whether pediatric patients have a higher risk than adults to develop PP-pericarditis and PP-RP. Observational studies on pediatric cohorts of RP have reported that PP cases account for about 10% of children and adolescents with PR (2). The risk of development of PP-pericarditis in children undergoing heart surgery might be particularly high after surgical repair of atrial septal defects (22); being reported to range between 10 and 28% (23–25), which is similar to what observed in adults.

### Secondary RP

#### RP in Systemic Autoimmune Disorders

Pericarditis is a frequent finding in patients with systemic autoimmune disorders (Table 2). In general, pericarditis in the setting of systemic autoimmunity might follow ether a chronic or an acute/subacute course reflecting the inflammatory activity of the underlying disease with multiple potential recurrences. On average, pericardial effusion tends to be larger but less symptomatic than that observed in idiopathic or viral pericarditis (26).

In the setting of systemic autoimmunity, the diagnostic work-up should screen for involvement of other heart structures, and consider specific complications related to immunosuppression such as lymphoproliferative diseases or infections.

##### Systemic lupus erythematosus (SLE) and connective-tissue diseases

SLE is the prototypic connective tissue disease, mainly affecting young women with a chronic-relapsing course. Up to 20% of SLE patients experience disease onset during infancy of adolescence (27). Heterogeneity in terms of disease severity and pattern of involvement is substantial, as SLE can involve most body tissues and organs. Prevalence of symptomatic pericarditis is about 25% in adults (28), and appears to be even higher in childhood-onset SLE (29). Although pericardial involvement is the most common cause of symptomatic heart disease in SLE, it is frequently asymptomatic and seldom results in major complications such as cardiac tamponade or pericardial constriction (26, 30, 31).

Idiopathic inflammatory myopathies, in particular dermatomyositis, are another connective tissue diseases that may affects pediatric patients (32–34). Although the skin and striated muscles are typical disease targets, pericardial involvement and pericarditis have been described, with lower frequency than SLE (26).

##### Systemic-onset juvenile idiopathic arthritis

Juvenile idiopathic arthritis (JIA) represent a heterogeneous group of diseases that globally accounts for most of childhood chronic rheumatic conditions (35). Systemic-onset JIA (SoJIA, also known as Still's disease) is characterized by polyarthritis, high-spiking fever with prominent acute-phase response, a fleeting pink skin rash, generalized lymphadenopathy, hepatosplenomegaly, and sometimes serositis. SoJIA pathogenesis include dysregulation in innate and adaptive immunity, thus presenting features of both autoimmune and autoinflammatory conditions. Cardiac involvement, predominantly pericarditis, is estimated to occur clinically in 10% of cases, and echocardiographic signs are observed in more than 30% of the cases (36, 37). Pericardial tamponade is uncommon, especially after therapy has been undertaken (38). Children and adolescents suffering from other forms of JIA might have asymptomatic pericardial effusion or a typically mild and benign pericarditis in up to 30 and 10% of cases, respectively (39).

##### Rheumatic fever (RF)

RF is a relapsing autoimmune disorder triggered by group-A streptococci infection in predisposed subjects. Inflammation typically affects the joints and the heart, and sometimes also the skin and the brain basal ganglia (40). Pathogenesis is due to molecular mimicry between streptococcal M protein and self-antigens. Pericarditis during RF is a sign of rheumatic carditis. Usually, it occurs at the initial episode, within 1 week after appearance of fever and arthritis (26), but sometimes is the presenting manifestation of RF or can present during relapses of RF (41). Pericarditis usually resolves without sequelae and does not require a specific management in addition to that for rheumatic carditis (antibiotics to eradicate streptococcal infection, salicilates, glucocorticoids, and management of valvulopathy and heart failure) (40).

##### Vasculitis

Pediatric vasculitides might uncommonly result in pericarditis. Despite being the most common pediatric vasculitis, Henoch-Schonlein purpura is not associated with pericarditis, as coexistence of the two has been reported only once (42).

Kawasaki disease is an acute, self-limiting muco-cutaneous febrile illness typically affecting children and characterized by small- and medium-sized arteries and frequently resulting in remodeling of coronary artery with stenosis or aneurysm. Pericardial effusion is frequently observed and is predictive of coronaritis and coronary artery remodeling (43). However, overt pericarditis is uncommon and typically uniphasic (44).

Takayasu arteritis is the prototypic large-vessel vasculitis (45, 46), and is the third most frequent pediatric vasculitis (47). Takayasu arteritis might be associated with pericarditis at disease onset or in the case of very high disease activity (48).

Behçet's disease can affect arteries and veins of variable size, skin and oral/genital mucosa, the bowel, the eye and the CNS (49). Five to ten percent of patients experience disease onset during childhood (50). Despite that Behçet's disease seldom affects the heart, pericarditis is the most common type of heart involvement (51).

ANCA-associated vasculitides (AAVs) are small-vessel vasculitides that can rarely affect pediatric patients. Although infrequently, pericarditis may be associated with active AAVs (26, 52).

##### Sarcoidosis

Sarcoidosis is the prototypic idiopathic granulomatous disease and affects multiple organs, mainly the lymph nodes and the lungs (53). Disease onset during childhood or adolescence occurs in <10% of subjects. Heart involvement might lead to cardiomyopathy with heart failure, conduction abnormalities, and arrhythmias. Mild to moderate pericardial effusion is frequently present during active sarcoidosis, while pericarditis is rare but can occur with a relapsing course (26).

##### Inflammatory bowel diseases

Inflammatory bowel diseases (IBDs) are chronic-relapsing inflammatory conditions, mainly represented by Crohn's disease and ulcerative colitis, that primarily affect the gut. Pediatric onset occurs in about 15–20% of subjects and portend a poorer prognosis (54). Extra-intestinal inflammatory involvement occurs in about a third of patients. Pericarditis is the most frequent extra-intestinal manifestation of IBDs, being reported in about 70% of subjects with cardiovascular complications (55). Pericarditis in the setting of IBD might follow a relapsing course and sometimes has onset before intestinal manifestations (56). Concurrent myocardial involvement is not rare and frequently depends on hypersensitivity to aminosalicylate therapy with mesalamine or sulfasalazine (55, 57–59).

#### RP in Autoinflammatory Disorders

Autoinflammatory diseases are a recently-recognized group of disorders in which inflammation results from dysregulated innate immunity rather than from autoimmunity. Many autoinflammatory diseases have been recognized as genetic disorders caused by mutations in key regulators of innate immunity. Although not being part of the typical disease features, pericarditis has been described in the setting of multiple autoinflammatory conditions, including Hyperimmunoglobulinemia D with periodic fever syndrome (HIDS) (60), NOD2-associated autoinflammatory syndrome (61), and chronic atypical neutrophilic dermatosis with lipodystrophy and elevated temperature (CANDLE) (62). On the contrary, two autoinflammatory conditions, namely Familial Mediterranean Fever (FMF) and TNF receptor-associated periodic syndrome (TRAPS), are closely related to IRP because of clinical similarities and a similar response to colchicine and anti-IL1 therapies such as anakinra.

##### Familiar Mediterranean Fever (FMF)

FMF is the most common monogenic autoinflammatory disease. It characterized by episodes lasting 1–3 days with fever, systemic inflammation, serositis and oligoarthritis (63). Overt peritonitis occurs in more than 90% of cases, pleuritis in about 40%, and pericarditis in about 5% (64–66). Subclinical pericardial disease might be more prevalent, as echocardiographic signs of pericardial effusion or thickening are present in up to 27% of patients (67). Pericarditis attacks during FMF often have a benign course without sequelae and tend to occur later in life (68).

##### TRAPS

TRAPS is a genetic condition characterized by febrile attacks lasting several days to weeks, associated with migratory erythema with underlying myalgia, ocular inflammation, arthralgia and/or arthritis, and serositis (69). Clinical and genetic heterogeneity of TRAPS is substantial, and attacks with isolated pericarditis have been described. Indeed, TRAPS mutations sometimes are identified in subjects with colchicine-refractory or steroid-dependent RP (70).

### RP Secondary to Other Pediatric Disorders

#### Camptodactyly–Arthropathy–Coxa Vara–Pericarditis (CACP) Syndrome

CACP syndrome is characterized by symmetrical, non-inflammatory arthropathy, synovial hyperplasia, congenital or early-onset camptodactyly, progressive coxa vara, and pericarditis. It is caused by mutations in the *proteoglycan 4* gene, which encodes lubricin, a lubricating glycoprotein of synovial fluid, articular cartilage and pericardium. Lubricin absence results in pericardial adhesions and fibrosis (71).

Non-inflammatory pericardial effusion is reported in up to 30% cases of CACP syndrome. Ascites and pleural effusions are uncommon. Pericarditis has a variable course, from a self-limiting condition to a chronic and constrictive evolution requiring surgical intervention.

### Diagnostic Specificities in the Pediatric Age

The ESC guidelines highlight that search for etiology is not mandatory at the first pericarditis attack in the absence of factors for a poor prognosis (Table 3) or of features suggestive of specific causes (1). However, RP represents a different scenario especially in children, where specific etiologies occur most frequently than in adults. In pediatric patients, high attention should be paid to identify genetic causes that might entail a specific management. Red flags for considering genetic screening for FMF or TRAPS are: (i) familiarity for RP or autoinflammatory diseases, (ii) a personal history of periodic fever, or (iii) colchicine-refractory or steroid-dependent RP.

**Table 3:** Prognostic factors predictive of complication or recurrence (1).

Major	Fever > 38^°^C Subacute onset Large pericardial effusion (>20 mm) Cardiac tamponade Lack of response to Aspirin or NSAID after ≥1 week of treatment
Minor	Myocardial involvement Immunosuppression Trauma Anticoagulant therapy

## Pathogenesis

IRP results from an interplay between environmental triggers, genetic predisposition and the immune system. The triggers of recurrence remain to be clarified. Recurrences are not associated with clinical features suggestive of a concurrent viral infection: it has been proposed that a tolerance break toward pericardial antigens at the first attack may pave the way for subsequent relapses. According to this view, dysregulated adaptive immunity and autoimmunity have a central role in IRP pathogenesis. This paradigm is supported by several observations, such as: (a) the occurrence of pericarditis in autoimmune diseases (72, 73), (b) the association of IRP with cardiac-specific and non-cardiac specific autoantibodies, such as antinuclear antibodies (ANA), anti-heart antibodies (AHA) and anti-intercalated disk antibodies (AIDA) (74), (c) the efficacy of steroids, of immunosuppressive agents targeting cellular immunity (e.g., azathioprine) and of immune-modulatory drugs such as intravenous immunoglobulin (75–79), and (d) the association of IRP with specific alleles in the human leukocyte antigen (HLA)–A, -C, and -DQB1 (80). Recently, this traditional view of IRP as an autoimmune disorder has been challenged by the discovery that dysregulated innate immunity is the driver of a group of inflammatory conditions, thus named “autoinflammatory diseases.”

**Figure 1:**
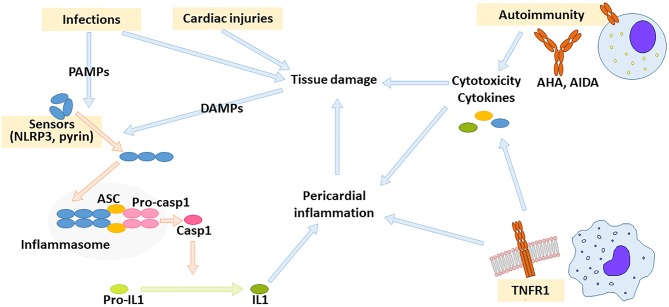
Drivers of pericardial inflammation. Autoimmunity against cardiac antigens as well as dysregulated innate immunity might result in pericardial inflammation. Innate immunity is activated by receptors for pathogen- or damage-associated molecular patterns (PAMPs and DAMPs, respectively). Crucial innate immunity pathways leading to pericardial inflammation depend on inflammasome activity and on TNF receptor-1 (TNFR1). The inflammasome is a multimolecular complex composed of sensor protein such as NLRP3 or pyrin (that self-assemble upon activation), stimuli such as NLRP3 or pyrin, adaptor proteins such as ASC, and pro-caspase-1. Upon inflammasome assembly, pro-caspase 1 releases active caspase 1, which can process pro-IL1 to active IL1. AHA: anti-heart antibodies, AIDA: anti-intercalated disc antibodies.

Innate immunity is triggered by germ-line encoded receptors expressed by multiple cell lines and results in an inflammatory response. These receptors recognize signals derived from microbial constituents (PAMPs, pathogen-associated molecular patterns) or from damaged tissue components (DAMPs, damage-associated molecular patterns). A crucial step in modulation of inflammation is the regulation of inflammasome activity (81). Inflammasomes are a family of multimeric complexes that activate caspase-1 and other proteases involved in inflammation. Inflammasomes are activated by specific sensor proteins belonging to the nucleotide-binding oligomerisation domain-like receptor (NLR) family. Upon stimulation, sensor proteins self-assemble into complexes (the inflammasomes) that recruit pro-caspase 1 (81). The adaptor protein ASC (Apoptosis Speck-like protein with a CARD domain) facilitates assembly and is typically required for efficient caspase 1 activation. One of the best-characterized inflammasomes derives from the sensor protein NLRP3 (NLR pyrine domain-containing-3). Recognition of multiple viral PAMPs and several DAMPs, such as urate crystals in gout and cholesterol in atherosclerosis (82–84), results in NLRP3 inflammasome activation and IL1 production (Figure 1). Gain-of-function mutations in the gene encoding pyrine, another sensor protein, may cause hyperactivity of pyrin inflammasome and result in FMF. A different inflammatory mechanism underlies TRAPS, which is caused by gain-of-function mutations in the receptor for Tumor Necrosis Factor (TNF)-α (69). Relatives of patients with TRAPS are frequently diagnosed with IPR, suggesting an overlap these conditions, especially for patients with low-penetrance mutations that may have a delayed disease onset or an incomplete TRAPS phenotype (85, 86).

The recognition that sterile pericarditis may derive from either dysregulated adaptive or innate immunity led to the proposal of an autoimmune and an autoinflammatory phenotype of IRP (87). However, distinction between the two is not is not always straightforward, due to the absence of specific biomarkers and to the strong overlap between innate and adaptive immunity, which are intrinsically co-entangled.

Similarly, the pathogenesis of post-cardiac injury pericarditis is poorly understood (16, 17). An autoimmune mechanism has been proposed to explain the onset of relapsing inflammation after tissue damage. Indeed, cardiac injury may result in the presentation of cardiac antigens in an immunogenic context. However, post-cardiac injury pericarditis has been observed in severely immunocompromised children after heart transplantation (88), suggesting that mechanisms alternative to autoimmunity can sustain inflammation. It logical to speculate that strong pathogenic similarities exist between IRP and post-cardiac injury RP, both resulting from dysregulated innate and/or adaptive immunity.

## Prognosis of Recurrent Pericarditis in the Pediatric Age

Prognosis of IRP is good in terms of mortality. No deaths were observed in children hospitalized for acute idiopathic or viral pericarditis in the pediatric health information system (PHIS) database (9). However, morbidity is significant due to multiple recurrences, medication side effects, or occurrence of complications such as cardiac tamponade, pericardial constriction and myocardial involvement. Prognostic factors associated with an increased risk of recurrences or of complications have been identified (Table 3), although they have been derived from unselected cohorts of patients with pericarditis and never validated in pediatric cohorts.

Cardiac tamponade typically occurs upon rapid accumulation of fluid in the pericardium, such as in the case of neoplastic pericarditis. Cardiac tamponade is much rarer (about 1–2% of cases) in idiopathic or in viral acute pericarditis (89). Cardiac tamponade complicates the first pericarditis attack more frequently than recurrences (90). Prevalence of tamponade in post-cardiac injury pericarditis is higher, being reported in 5–20% of subjects (19, 91, 92). Cardiac tamponade warrants prompt pericardiocentesis, but subsequent clinical course is similar to uncomplicated pericarditis (75).

Pericardial constriction is a hemodynamic condition due to hampering of diastolic filling of ventricles by a fibrotic and inextensible pericardium. Occurrence of pericardial constriction is rare (1–2% at 6 years of follow-up) in patients with idiopathic or viral pericarditis, relatively infrequent (2–13%) in those with post-cardiac injury pericarditis or pericarditis in the settings of other systemic autoimmune disorders, and frequent (20–30%) in the case of tubercular, purulent or post-actinic pericarditis (91, 93). Pericardial constriction has never been reported in IRP (90, 94). Severe constriction requires surgical pericardiectomy, although medical therapy with NSAIDs sometimes results in improvement in the case of viral, idiopathic or immune-mediated pericarditis (95, 96).

Myocardial involvement during acute pericarditis occurs in about 15% of adult subjects (75) and up to 35% of pediatric patients (97). Myocardial inflammation is revealed by increased troponin levels or by myocardial inflammation/fibrosis at cardiac magnetic resonance (MR). It has been proposed to distinguish two conditions with concomitant pericardial and myocardial inflammation: myopericarditis and perimyocarditis. The former is characterized by predominant pericardial inflammation extending from the epicardial fat to the myocardium. Myopericarditis has clinical features highly similar to pericarditis and usually follow a benign course with a reduced risk of recurrences or cardiac tamponade. Conversely, perimyocarditis patients tend to have less intense pericardial pain and elevation of acute-phase reactants but prominent myocardial inflammation with regional or global reduction in systolic function (98). Most of these patients recover a normal left ventricular function after the resolution of the attack.

## Role of Imaging

In patients with cardio-respiratory symptoms, chest X-ray may reveal concomitant conditions or alternative diagnosis to pericarditis, or identify pleural and pericardial effusion. Thus, chest X-ray is the first imaging technique usually performed, although it is inaccurate in the quantification of the amount of pericardial fluid, and it is unable to assess the cardiac function and to differentiate among the various etiologies of pericarditis.

Echocardiography is the technique of choice, it is widely and rapidly available, and can be repeated during follow-up. It allows to study the dimensions and functions of cardiac chambers and valves. Signs suggestive of active pericarditis are pericardial effusion and hyperechoic pericardium. In addition, echocardiography may quantify pericardial effusion and reveal potential complications such as cardiac tamponade, systolic dysfunction as well as signs of pericardial constriction.

In selected cases, cardiac MR might be complementary to echocardiography, thanks to its ability to (i) provide an excellent depiction of cardiac and pericardial morphology together with a good quantification of pericardial effusion and ventricular or valvular function, (ii) characterize myocardial and pericardial tissue in terms of inflammation, edema and fibrosis, and (iii) reveal findings suggestive for concomitant diseases, such in the case of autoimmune diseases, large-vessels vasculitis, thoracic lymphadenopathies, or cardiac tumors. Acquisition protocols specific for pediatric patients may require to take into account small heart dimension and fast heart rates and to ensure reliable breath-holding (99). Briefly, specific signs of active pericardial inflammation at MR are pericardial edema (hyperintensity on T2-weighted short-tau inverted recovery [STIR] sequences) and pericardial late gadolinium enhancement (LGE, meaning pericardial signal enhancement in T1-weighted sequences obtained 10 min after contrast medium administration). Pericardial thickening (>4 mm) is not considered a specific sign of active pericarditis because it can be observed in multiple pericardial disorders including pericardial constriction or neoplasms. Cardiac MR might also revel concomitant myocarditis. It is proposed that the kinetic of gadolinium ingress and egress differs between the pericardium and the myocardium. Accordingly, active myocardial inflammation is heralded by edema and early gadolinium enhancement (observed on T1-weighted sequences obtained after about 2 min after contrast medium administration). Differently from the pericardium, myocardial late gadolinium-enhancement reveals fibrosis (75).

Given the specificities of cardiac MR, its indications in the setting of pericarditis are: (i) atypical cases, to confirm pericarditis or identify alternative diagnoses, (ii) pericardial constriction or myocardial involvement, and (iii) RP associated to specific etiologies such as large-vessel vasculitis (100), and (iv) need to tailor therapy according to the intensity or persistence of pericardial inflammation (101).

Computed tomography (CT) is another complementary technique. Similarly to MR, CT provides good anatomic images and may quantify pericardial thickness, the volume of pericardial effusion or the presence of localized effusion. In addition, CT can assess the presence of pericardial calcifications and define the attenuation values of the pericardial fluid which might be of help in the diagnostic workup: high values suggest hemorrhage, intermediate values exudative effusions and low values trasudative effusions (102). CT is particularly useful in the initial diagnostic work-up to exclude specific etiologies including malignancies and tuberculosis, and in the preoperative planning of pericardiectomy.

## Management of Recurrent Pericarditis in the Pediatric Age

Management of pediatric patients with RP is derived from the experience with adult patients: it includes medical and interventional therapies, and lifestyle recommendations. After correct diagnostic workflow, therapy should be targeted to the underlying etiopathogenesis as much as possible, aiming at inducing remission and preventing recurrences and complications. Primary prevention of pericarditis (e.g., in the setting of cardiac surgery) is beyond the scope of this review. Available evidence has not suggested important differences in the management of recurrences and remission phases of post-cardiac injury RP and IRP.

### Management of Acute Attacks

Admission should be considered in presence of severe pain or predictors of poor prognosis (Table 3) (1). Acute pericarditis should be treated with high-dose NSAIDs in combination with colchicine, except for specific etiologies requiring alternative treatments or for refractory subjects (1). High-dose aspirin is generally not used in pediatric patients, due to concerns about the risk of Reye syndrome. In this setting, ibuprofen (30–50 mg/kg daily, divided every 6–8 h) or indomethacin (2 mg/kg daily, divided every 6–12 h, Table 4) are valid options. Intravenous administration might be useful to achieve rapid pain control in hospitalized patients.

**Table 4:** Medical therapy for recurrent pericarditis in children.

**Agent**	**Starting dose**
NSAID	
Ibuprofen	30–50 mg/kg daily, divided every 6–8 h
Indomethacin	2 mg/kg daily, divided every 6–12 h
Colchicine	0.5 mg/day before 5 years of age
	1–1.5 mg/day after 5 year of age
Anakinra	1–2 mg/kg daily up to 100 mg daily subcutaneously
	Start tapering not before 3–6 months of remission
Prednisone	0.5–2 mg/kg daily
IVIG	400–500 mg/kg for 5 days (repeatable once a month)

Colchicine is an anti-gout medication that is also active for FMF (103) and pericarditis. Colchicine concentrates within leukocytes (especially granulocytes) and inhibits microtubule assembly, thus limiting cell motility, phagocytosis, and degranulation. Moreover, colchicine downregulates NLRP3 inflammasome by antagonizing caspase-1 activity and potential triggering factors (e.g., P2X2 and P2X7 channels and Reactive Oxygen Species) (104). Gastrointestinal intolerance is the most common side effect, it is dose-dependent and reported in about 5–10% of adults (5, 12, 13), although children might tolerate higher per kilo doses than adults. Studies have shown that colchicine hastens the response to treatment and decreases the risk of recurrences of about 50% (105). Moreover, colchicine reduces the risk of pericardial constriction in PP pericarditis (91). Unfortunately, little evidence is available about colchicine in pediatric patients with RP (106): a recent observational study reported a 65% reduction of recurrences (2). Moreover, colchicine therapy in pregnant women with IRP raised no concerns about fetal toxicity (107). Despite this encouraging data about safety and efficacy in children with RP, colchicine remains underprescribed in the pediatric population (9, 108).

Currently, steroids are used as second line therapy in adults with sever acute pericarditis or with colchicine-refractory RP: they are rapidly effective but they favor recurrences and steroid-dependence, especially if used at high doses (109, 110). In children, steroids may cause growth retardation, acne, *striae rubrae*, and predisposition to osteoporosis. Therefore, steroids should be avoided as much as possible in pediatric IRP by means of the following recommendations: (i) to use NSAIDs at the maximum tolerated does or (ii) to intravenously administer NSAIDs in hospitalized patients, and (iii) to consider the use of anti-IL1 therapy after failure of high dose NSAIDs combined with colchicine. Alternatively, steroids should be started at the lowest effective dose, and slowly tapered after remission has been obtained.

Anti-IL1 therapy with anakinra (1–2 mg/kg daily subcutaneously) is the most important advance in the last decade for the field (111). Anakinra is efficacious for multiple autoinflammatory diseases including FMF. Thus, it has been initially used in children with refractory IRP. Subsequent experience including a clinical trial has shown that anakinra has spectacular effects in refractory or steroid-dependent IRP with raised acute-phase reactants (2, 15, 112–114). Children with IRP frequently have an “autoinflammatory phenotype” (2) that is particularly responsive to anakinra. Anakinra has a very good safety profile, due to its short half-life and low risk of infections and of reactivation of tuberculosis. Severe reactions are rare, but injections site-reactions are frequent in the first month of treatment, and then disappear (111). Thus, anakinra should be considered as a second line agent for children and adolescents with IRP with raised acute-phase reactants that is refractory to NSAIDs and colchicine.

Intravenous immunoglobulins (IVIG) are used for refractory cases at the dose of is 400–500 mg/kg for 5 days, potentially repeatable after 1 month (2, 77–79). A systematic review about IVIG for RP including 19 adults and 11 pediatric patients, showed good efficacy and safety of IVIG (79). The main limitations of IVIG are costs, the intravenous administration and the administration schedule. Since anti-IL1 therapy has become available for IRP, the role of IVIG is mainly limited to patients with autoimmune features.

RP in the setting of specific etiologies should be treated accordingly to the underlying disease. Specifically, RP in the setting of FMF should be treated with colchicine and anti-IL1 therapies (anakinra, rilonacept, canakinumab) for refractory cases (63). In RP due to TRAPS patients should be treated with NSAIDs, steroids and anti-IL1 treatments or etanercept (115, 116). SLE-associated RP should be treated with a combination of hydroxychloroquine, a brief steroid course and immunosuppressive agents such as azathioprine and mycophenolate mofetil (117, 118).

### Prevention of Recurrences and Other Complications

After complete remission (absence of symptoms and of raised acute-phase reactants) has been achieved, therapy can be slowly tapered, reducing a single class of drug at a time. Steroids and high-dose NSAIDs are tapered first. Table 5 shows the steroid-tapering schedule that we follow in pediatric patients with IRP, directly derived from recommendations for adult patients (1). Recurrences are particularly frequent when steroids are tapered below 0.2 mg/kg^*^day of prednisone-equivalent, and small decrements are advisable every at least 2–6 weeks (e.g., reductions of 1–2.5 mg on alternate days). Tapering of anakinra should not start before 3–6 months of sustained remission, and should be performed very slowly due to high risk of recurrences.

**Table 5:** Tapering of glucocorticoids in children.

Daily dose^*^	**Tapering**
>0.7 mg/kg	0.14 mg/kg^*^day every 1–2 weeks
0.35–0.7 mg/kg	0.7–14 mg/kg^*^day every 1–2 weeks
0.2–35 mg/kg	0.035 mg/kg^*^day every 2–4 weeks
<0.2 mg/kg	0.017–0.035 mg/day every 2–6 weeks

* *Doses are expressed as prednisone-equivalents*.

Slow-acting medications including hydroxicloroquine or immunosuppressive agents such as azathioprine, mycophenolate or cyclosporine (2, 76), have been proposed as 3rd or 4th line medications to prevent recurrences in the cases of refractoriness to anakinra or of features suggestive of autoimmune pathogenesis.

With the exception of acute-phase reactants, we lack biomarkers to guide therapeutic tapering during remission and to predict future exacerbations. Cardiac MR has been proposed at this purpose, although its use in young children might be troublesome because of durations of acquisition and capability to ensure adequate breath holds.

### Interventional Therapy for Relapsing Pericarditis

Cardiac tamponade requires emergent pericardiocentesis to restore adequate heart filling. Selected cases of acute pericarditis (mainly at the first episode) might require diagnostic pericardiocentesis if specific etiologies are suspected, including neoplasms or bacteria.

Surgical pleuropericardial window is another options that might be considered for subjects at risk of recurrent pericardial tamponade. In the case of severe or chronic pericardial constriction or of RP refractory to multiple therapeutic lines, surgical pericardiectomy might be of help. However, evidence about pericardiectomy for refractory RP is limited to adults (119), and this procedures should be considered only as an *extrema ratio* in pediatric patients.

### Lifestyle Recommendations

Based on expert recommendations, children should avoid physical activity after acute attacks until the resolution of symptoms and acute-phase reactants. Moreover, resumption of competitive sports should occur not before 3 months after complete remission of pericarditis. In case of frequent recurrences avoidance of physical activity in children in our opinion is less stringent, since it is important to allow a normal or near-normal life in these children; the focus should be on a therapy able to control the disease more than on restriction of physical activity.

Exacerbations of SLE or autoinflammatory diseases are sometimes associate with specific triggers, including sunlight for SLE, and exercise, local injury, infection, cold exposure, emotional stress, surgery and hormonal changes for FMF and TRAPS. Patients with RP associated with these condition should be advised to avoid potential triggers, especially if they have been involved in previous disease flares.

The recommended vaccination schedule (120) may not require changes for most children with RP. Prevention of recurrences by influenza vaccination is not demonstrated, reflecting that influenza is a rare trigger of RP. Subjects treated with immunosuppressive agents might benefit from all available inactivated vaccines, although immunogenicity might be reduced and additional administrations be required. On the contrary, a careful balance of the degree of immunocompromised, the risk of natural exposure and the availability of non-live alternatives, may be required for attenuated vaccines.

## Conclusions

RP in children and adolescences has significant morbidity. Multiple potential causes exist, although most of them are related to either autoimmune or autoinflammatory mechanisms. Recent advances allow to manage RP effectively in almost all patients. However, a careful diagnostic work-up and a correct therapeutic algorithm are required to maximize efficacy while limiting avoidable costs and side effects.

## Author Contributions

ET, TG, AB, and RC designed the study and drafted the manuscript. All authors agree to be accountable for the content of the work.

### Conflict of Interest

AB declares unrestricted research grants by ACARPIA e SOBI. The remaining authors declare that the research was conducted in the absence of any commercial or financial relationships that could be construed as a potential conflict of interest.

The reviewer GL declared a past collaboration with one of the authors AB to the handling editor.
